# Puerperal Fever in London in 1906

**Published:** 1908-01-18

**Authors:** 


### Gynæcology.

### PUERPERAL FEVER IN LONDON IN 1906.

### THE WORKING OF THE MIDWIVES ACT.

In the report of the Medical Officer of Health of the County of London for 1906 there are some interesting figures dealing with obstetrical practice. The number of deaths registered as due to puerperal sepsis was 187, an increase of four over the previous year. The notifications of puerperal fever were only 298, and the total number of cases which came under official notice was 366. It might therefore be concluded that the death rate of puerperal fever was $\frac{187}{366}$, or over 50 per cent. Now puerperal fever, according to the Registrar and the Royal College of Physicians, includes septicæmia, sapræmia, pyæmia, peritonitis, and metritis, occurring in connection with parturition. It is obvious that the explanation of the very high death rate just deduced is that very many cases go unnotified; it would probably be no overstatement to say that in private practice very few cases indeed are notified unless there is a probability of a fatal issue. That many cases do go unnotified is proved by the fact that out of the 187 fatal cases 68 were of this nature, and it is safe to conclude that of milder cases there were a much larger number than appears from the report. The Medical Officer recognises this when he says, with reference to the official definition of puerperal fever, that "a more limited interpretation of the term appears to be often adopted for purposes of notification." More than that, it is beyond question a fact that a certain number of fatal cases are ascribed to other conditions, so that even the death rate appears less than it really is. The death rate per 1,000 births from puerperal fever in 1906 was 1.50, which is higher than that of five out of the last fifteen years, and distinctly above what it ought to be.

Of the 366 cases of puerperal fever 90 occurred in the practice of certified midwives, with 38 deaths; this may be taken to show that, as might be expected, fewer non-fatal cases escape notification in the practice of midwives than in the community at large. The history of the 90 cases shows that 39 had some abnormality of the puerperium, such as perineal rupture, retained or adherent placenta, antepartum illness or hæmorrhage, or instrumental delivery.

The report shows that about 2 per cent. of the births attended by midwives are still, but reasons are advanced for believing that not all of them are notified, and that the true percentage is a little over three. The total number of births recorded by some 212 midwives, including most of these with large practices, is 30,749: of these 212 women, 22 attended between 200 and 300 births each, and an equal number attended upwards of the latter figure. Probably most, if not all, of these are midwives on the extern staff of maternity charities, where medical help is easily to be had; for it is also recorded that five midwives sent in statutory requests for assistance fifty or more times each, and twenty others on over twenty occasions.

The number of midwives on the roll during the year was 2,350, of whom only 184 obtained that privilege by having been in practice before the Act of 1902. Out of the 2,350, however, but 490 gave notice of their intention to practise, and this number is said to include the greater part of the 184; so that a considerable percentage of practising midwives are still more or less untrained. Some 200 midwives never called in medical aid, from which it may be assumed that they were not in regular practice.

We do not gather exactly how many births were attended by enrolled midwives, and by them only, in London during the year. The 212 who sent in returns attended, as has been mentioned, over 30,000 cases, a quarter of the total births in the county, and if the figures of the remaining 78 in regular, and of the 200 in occasional, employ could be added, the proportion would doubtless be not far short of 30 per cent. It is interesting to note that of the 187 deaths from puerperal sepsis 38 were those of women delivered by certified midwives (including four of instrumental delivery); thus at the highest computation the midwives are concerned in but 20.3 (more fairly to be reckoned as 18.2) per cent. of the mortality from puerperal fever, as compared with about 30 per cent. of the total births. It is, of course, difficult to compare these figures with those of the cases attended by the medical profession, for the report affords no information as to how many cases are attended by uncertified women. It may be remarked, however, that 75 of these are known to the authorities as in regular practice, and that three inquests were held upon their patients, though this by no means represents the whole of the mischief which they do. In favour of the enrolled midwives it is to be remembered that of their cases a large number are the beneficiaries of one or other of the maternity charities, and that the remainder pay fees averaging only 7s. 6d. This means that the midwives attend a large number of the poorest mothers in London, and in their own homes where the prevailing conditions are most unfavourable to the efficient prophylaxis of puerperal fever. The figures which are quoted in, or to be deduced from, this report are therefore very greatly to their credit, and prove the immense value of their services to the community.

Moreover a death from puerperal fever in the practice of a midwife is practically certain to be registered as such, while a certain number of others are classified in the returns under other headings. It is, therefore, in view of the facts and figures quoted, no very great credit to the practising obstetricians of the Metropolis that, notwithstanding the Midwives Act and the excellent results obtained by those licensed under it, the general mortality from the septic complications of childbirth remains almost stationary. The reproach is one which has been levelled before, and the figures for 1906 certainly afford justification for it.

